# Prognostic Re-evaluation of Antinuclear Antibodies in Childhood Immune Thrombocytopenia

**DOI:** 10.1055/a-2938-6571

**Published:** 2026-09-17

**Authors:** Zeinab Muse, David E. Schmidt, Katja M.J. Heitink-Pollé, Leendert Porcelijn, Kyra A. Gelderman, Marrie C.A. Bruin, C. Ellen Van der Schoot, Gestur Vidarsson, Masja De Haas, Rick Kapur

**Affiliations:** 1 Pediatric Coagulation 167725 Astrid Lindgren Children’s Hospital, Karolinska University HospitalStockholmStockholm CountySweden; 2 Department Research 1193 Sanquin Blood Supply FoundationAmsterdamNoord-HollandNetherlands; 3 Landsteiner LaboratoryAmsterdam University Medical Center, University of AmsterdamAmsterdamNetherlands; 4 Princess Maxima Center for Pediatric OncologyUtrechtNetherlands; 5 Department of Pediatric Hematology 8124 University Medical Centre UtrechtUtrechtNetherlands; 6 PurSang DiagnosticsAmsterdamNoord-HollandNetherlands; 7 Department of Immunology 6993 Erasmus Medical CenterRotterdamZHNetherlands; 8 Department of Biomolecular Mass Spectrometry and Proteomics 534214 Utrecht Institute for Pharmaceutical SciencesUtrechtNetherlands; 9 8125 Bijvoet Center for Biomolecular Research, Utrecht UniversityUtrechtNetherlands; 10 Department of Hematology 4501 Leiden University Medical CenterLeidenZHNetherlands; 11 Department of Medical AffairsSanquinAmsterdamNetherlands

**Keywords:** autoantibodies, pediatrics, immune thrombocytopenia, laboratory assays, prognosis

## Abstract

**Background** In the autoimmune bleeding disorder childhood immune thrombocytopenia (ITP), antinuclear antibodies (ANA) have been suggested as potential biomarkers for predicting chronic ITP. Unfortunately, published evidence remains conflicting and testing is not currently recommended in clinical routine.

**Aim** This study aimed to validate the prognostic association of ANA with chronic ITP in newly diagnosed childhood ITP.

**Methods** Data from 176 children with newly diagnosed ITP and a platelet count ≤20 × 10 ^9^ /L enrolled in the Treatment With or Without IVIg in Kids with ITP (TIKI) randomized controlled trial were available. ANA testing was performed on sera obtained within 72 h of diagnosis, before immunomodulatory treatment. Children were followed for 12 months to assess chronic ITP development, intravenous immunoglobulin (IVIg) treatment response, and spontaneous recovery.

**Results** ANA were detected in 36/176 (20%) children. Chronic ITP developed in 21/176 children; 4/36 (11%) of ANA-positive versus 17/140 (12%) of ANA-negative patients (OR 0.90; 95% CI: 0.25–2.65). No differences were observed in platelet counts, spontaneous recovery, or IVIg responses when comparing children with and without ANA. ANA provided no additional prognostic value beyond the Childhood ITP Recovery Score, a clinical prediction rule for recovery from ITP. ANA were not associated with anti-platelet antibodies by platelet immunofluorescence (PIFT) or monoclonal antibody-immobilization of platelet antigens (MAIPA).

**Conclusion** ANA testing at the time of diagnosis was not helpful to predict chronic ITP, IVIg treatment responses, or spontaneous recovery among children with newly diagnosed ITP and a platelet count ≤20 × 10 ^9^ /L. Future studies may evaluate whether ANA testing may be of value when performed at a later point in time.

## Introduction

Immune thrombocytopenia (ITP) is an acquired autoimmune bleeding disease, with complex underlying pathophysiology that results in increased platelet destruction and decreased platelet production. 1 2 Childhood ITP evolves differently from adult ITP. While most children recover spontaneously, around 40% display persistent ITP (thrombocytopenia >3 months), and 10 to 25% develop chronic ITP (>12 months). 3 4 Persistent and chronic ITP are associated with reduced health-related quality of life and increased overall morbidity. 5 6 First-line treatment aims to dampen the autoimmune response, using corticosteroids or IVIg.

A key clinical challenge is identifying children at risk of developing persistent and chronic ITP at the time of diagnosis, which may inform family counseling, guide additional diagnostic efforts, and allow for individualized treatment. To predict development of persistent/chronic ITP at the time of diagnosis, clinical features may be used. The externally validated Childhood ITP Recovery Score 7 predicts this with moderate certainty, based on older age at diagnosis, insidious disease onset, female sex, lack of preceding infection, and higher platelet counts. To improve such clinical predictions, biomarkers are considered to hold strong potential, but no specific markers are currently clinically established to predict which children with acute ITP will progress to persistent or chronic ITP.

One suggested biomarker related to the autoimmune nature of ITP is antinuclear antibodies (ANA). In a meta-analysis of three retrospective studies, children with newly diagnosed ITP and ANA may be at moderately increased risk of developing chronic ITP with an odds ratio of 2.9 (95% CI: 1.6–5.2). 8 However, recent data question this association. 9 Conceptually, ANA target antigens found in the cell nucleus and are commonly detected in autoimmune diseases, and there is also an association between ITP and systemic lupus erythematosus and Sjögren’s syndrome. 10 Smaller studies detected ANA among 9 to 44% of children with ITP, varying significantly between study cohorts due to differences in inclusion criteria, blood sampling delay relative to diagnosis, and previous immune treatments. 11 12 13 In sum, the prevalence and association of ANA with chronic ITP have not been confirmed in a larger and unselected population, and uncertainty remains regarding the relevance and timing of ANA testing among children with newly diagnosed ITP.

Here we used data from a randomized controlled trial as a platform to validate the prognostic association of ANA with chronic ITP among children with newly diagnosed ITP. Secondary outcomes were the association of ANA with response to IVIg or spontaneous recovery during 12 months of observation. We also evaluated the added value of ANA testing to risk stratification by clinical characteristics with the Childhood ITP Recovery Score.

## Methods

We analyzed clinical data and serum samples from the Treatment With or Without IVIg in Kids with ITP (TIKI) multicenter randomized controlled trial. 14 Children between the ages of 3 months and 16 years with a diagnosis platelet count of ≤20 × 10 ^9^ /L were eligible, a platelet count that includes approximately 80% of newly diagnosed childhood ITP. 15 Participants were randomized to receive either a single dose of 0.8 g/kg IVIg or careful observation. Key exclusion criteria were the presence of severe bleeding at diagnosis (grade 4 or above in the Buchanan score), contraindications to IVIg, immunomodulatory drugs within 1 month of diagnosis, and signs of an alternative cause of thrombocytopenia or hereditary thrombocytopenia. All parents and patients aged 12 years and above gave written informed consent before inclusion. Baseline characteristics were obtained at diagnosis. Serum samples were obtained at inclusion (maximum 72 h after diagnosis; no ITP treatments given). Indirect platelet immunofluorescence (PIFT) and monoclonal antibody-immobilization of platelet antigens (MAIPA) tests for antiplatelet antibodies were performed as previously described. 16 17 The study was approved by the Institutional Review Board of University Medical Center Utrecht and conducted in accordance with the Declaration of Helsinki.

Reactivity with nuclear antigens was detected by indirect immunofluorescence for nuclear staining of Hep-2 cells in a clinical diagnostic department (Department of Immunochemistry, Sanquin Diagnostics, Amsterdam, The Netherlands). In brief, Hep-2 cells were incubated for 30 minutes at a serum dilution of 1:40, washed, and evaluated for nuclear staining using fluorescently labeled anti-human IgG, as described previously. 18 The Hep-2 nuclear staining fluorescence intensity was evaluated by microscopy and dichotomized into positive (strongly positive [+++], positive [++], slightly positive [+/−]) and negative samples (dubious and negative). Among healthy adult controls, approximately 5% show positive ANA with this assay (Sanquin Diagnostics), comparable to a 1:80 dilution in other settings. Where indicated, further titration was performed at dilutions of 1:160 and 1:320. For sensitivity analysis of ANA titer, high-titer was defined as reactivity at ≥1:160; all other samples (ANA-negative, weakly positive at screening and not titrated, or no reactivity at 1:160) were classified as negative/low-titer.

Statistical analyses were performed in R version 4.4.0 (R Core Team). Differences in categorical variables were tested with a chi-squared test, and continuous variables with the Wilcoxon rank-sum test. The association between ANA and chronic ITP was evaluated by logistic regression. Recovery from ITP during clinical follow-up was assessed by survival analyses, with the additional value of ANA testing over the Childhood ITP Recovery Score tested by a Cox proportional hazard model, and illustrations with Kaplan-Meier plots. Statistical significance was defined at *p* < 0.05.

## Results

Sufficient serum was available for 176/200 children enrolled in the TIKI trial ( Fig. 1 ). The 24 children without available serum did not differ in age, sex, platelet count, or chronic ITP rate from those with available samples (data not shown). ANA (Hep-2 cell nuclear staining fluorescence) was positive in 36/176 (20%; 95% CI: 15–26%) children at diagnosis, compared with approximately 5% of healthy adults with this assay. Baseline characteristics stratified by ANA status are shown in Table 1 . There was a non-significant trend toward ANA being more common in children above 10 years of age and females. Children with positive ANA tended to be older (median age 5.8 vs. 3.8 years, *p* = 0.18) and were more often female (61% vs. 43%, *p* = 0.08), though these differences were not statistically significant. Platelet counts, preceding infection rates, and leukocyte parameters were similar between groups.

**Fig. 1:**
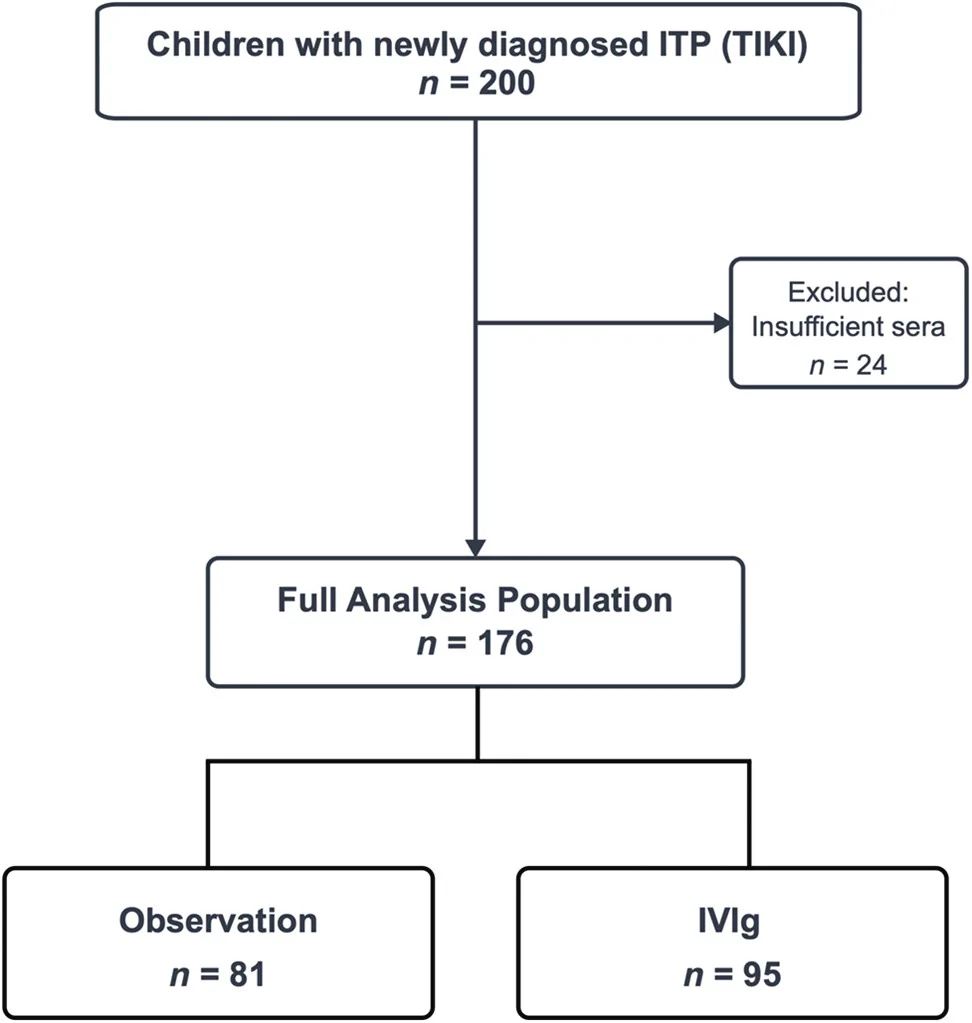
Study flowchart. ITP, immune thrombocytopenia; TIKI, Treatment With or Without IVIg in Kids with ITP.

**Table 1:** Baseline characteristics of the study population.

	Total ( *N* = 176)	ANA negative ( *N* = 140)	ANA positive ( *N* = 36)	*p* -Value
IVIg	95 (54)	77 (55)	18 (50)	0.73
Age at diagnosis, years	3.9 (2.5–7.6)	3.8 (2.4, 7.5)	5.8 (2.8, 10.5)	0.17
<1 year	7 (4.0)	5 (3.6)	2 (5.6)	0.28
1–9 years	135 (76.7)	111 (79.3)	24 (66.7)	
≥10 years	34 (19.3)	24 (17.1)	10 (27.8)	
Female	82 (46.6)	60 (42.9)	22 (61.1)	0.08
Duration of symptoms, days	3 (2, 7)	3 (2, 7)	4 (2, 11)	0.18
Diagnosis platelet count, ×10 ^9^ /L	6.0 (3.0, 9.3)	6.0 (3.0, 10.0)	6.0 (2.8, 8.3)	0.84
Preceding infection	94 (54.0)	73 (52.9)	21 (58.3)	0.69
Preceding vaccination	8 (4.6)	7 (5.1)	1 (2.8)	0.89
Mucosal bleeding	72 (41.1)	55 (39.6)	17 (47.2)	0.52
Leukocytes, ×10 ^9^ /L	8.2 (6.5, 10.7)	8.3 (6.4, 10.7)	8.1 (6.7, 10.4)	0.94
Lymphocytes, ×10 ^9^ /L	3.8 (2.0, 7.0)	3.9 (2.7, 5.0)	3.50 (2.7, 4.9)	0.53

Abbreviations: ANA, antinuclear antibody.

Notes: Data are *n* (% of column) or median (IQR). Preceding infection/vaccination within 28 days of the diagnosis.

*p* -Value for difference between positive and negative ANA groups.

Of 176 children assessed at diagnosis, 21 developed chronic ITP (platelet count <100 × 10 ^9^ /L at 12 months). Complete follow-up until 12 months or recovery was available for all 176 patients. The prevalence of chronic ITP among children with ANA at the time of diagnosis was 4/36 (11%; 95% CI: 1–21%), compared with 17/140 in patients without ANA at the time of diagnosis (12%; 95% CI: 7–18%). The odds ratio of a positive ANA test at diagnosis and development of chronic ITP was 0.90 (95% CI: 0.25–2.65), indicating that ANA was not associated with chronic ITP. Both patients randomized to IVIg and observation showed the same chronic ITP rate among children with ANA (2/18 in the IVIg group and 2/18 in the observation group).

As a secondary outcome, to evaluate whether ANA predicts treatment response or spontaneous recovery, we compared platelet trajectories and recovery rates during 12 months of follow-up. Median platelet counts and complete recovery rates over time were similar between ANA-positive and ANA-negative children, regardless of treatment allocation ( Fig. 2 ; details in **Supplementary Table S1** (online only)). By 12 months, complete recovery (platelet count ≥100 × 10 ^9^ /L) occurred in 32/36 (89%) ANA-positive and 123/140 (88%) ANA-negative children.

**Fig. 2:**
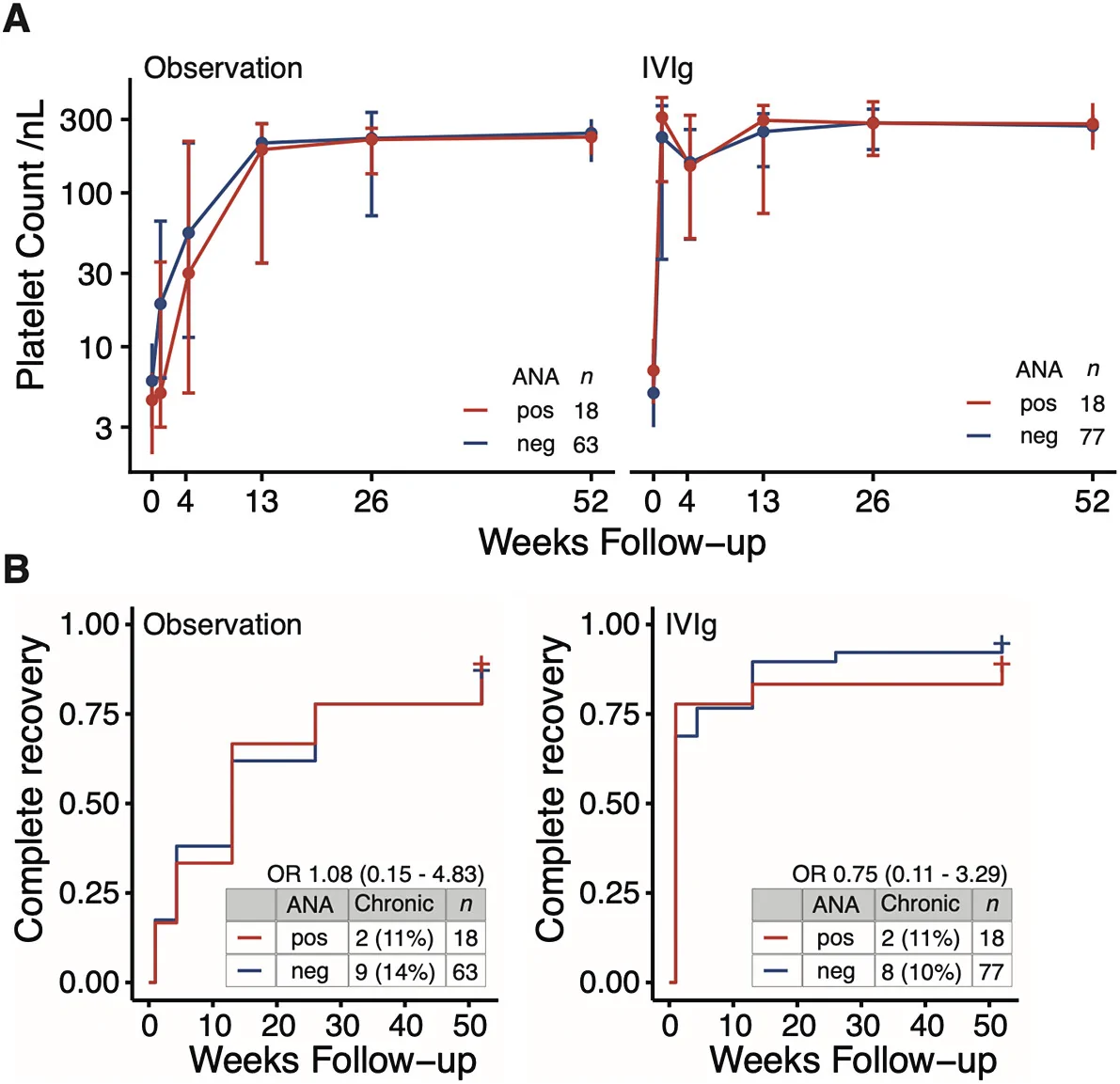
Antinuclear antibodies (ANA) and prognosis in newly diagnosed immune thrombocytopenia (ITP). ( **A** ) Median (IQR) platelet count evolution during 1 year after the diagnosis, stratified by ANA status. ( **B** ) Kaplan-Meier plot for longitudinal recovery from newly diagnosed ITP. Complete recovery was defined with a platelet count ≥100 × 10 ^9^ /L during careful observation (left panel) and after IVIg administration (right panel). Odds ratio (OR) and 95% CI of a positive ANA test at the time of diagnosis for the odds to develop chronic ITP versus recovery.

Previous studies suggested that ANA might have prognostic value specifically in female adolescents without preceding infection and a more insidious onset of ITP. We therefore evaluated whether ANA adds value after initial risk stratification with the Childhood ITP Recovery Score, 7 a validated clinical prediction rule which includes these clinical features. After adjusting for the Childhood ITP Recovery Score and treatment allocation, ANA status offered no improved prediction of complete recovery (adjusted hazard ratio 1.09, 95% CI: 0.73–1.63; *p* = 0.67; adjusted for score and treatment). Similarly, ANA showed no prognostic value within any risk stratum of low-, intermediate-, or high-risk subgroups of the Childhood ITP Recovery Score. These findings indicate that ANA testing provides no incremental prognostic information beyond readily available clinical characteristics.

To explore whether ANA might reflect broader autoimmune dysregulation, we examined the association of ANA with antiplatelet antibodies measured by PIFT or MAIPA for either IgG or IgM. We found no association of ANA with antiplatelet antibodies ( Table 2 ).

**Table 2:** Association of antinuclear antibodies (ANA) and antiplatelet antibodies at the time of diagnosis.

		ANA negative	ANA positive	*p* -Value
PIFT ( *N* = 176)	Positive	7 (5.1)	3 (8.3)	0.43
	Negative	131 (94.9)	33 (91.7)	
MAIPA IgM ( *N* = 163)	Positive	76 (59.8)	24 (66.7)	0.58
	Negative	51 (40.2)	12 (33.3)	
MAIPA IgG ( *N* = 174)	Positive	13 (9.4)	4 (11.4)	0.75
	Negative	126 (90.6)	31 (88.6)	

Abbreviations: MAIPA, monoclonal antibody-immobilization of platelet antigens; PIFT, platelet immunofluorescence.

Note: Antibodies measured by indirect PIFT and IgM or IgG MAIPA test against GP Ib/IX, GP IIb/IIIa, and GP V. *p* -Value given for a Fisher’s exact test or chi-squared test, as appropriate.

As confirmatory testing, 14 ANA-positive samples were further titrated at 1:160 and 1:320 dilutions, consistent with a dose-dependent titration pattern ( **Supplementary Table S2** (online only)). We performed a subgroup analysis for the children showing high-titer ≥1:160 ANA positivity, observed among 6 of 176 children (3%). Chronic ITP developed in 2/6 (33%) high-titer versus 19/170 (11%) negative/low-titer children (OR 3.97; 95% CI: 0.33–29.6; Fisher’s exact *p* = 0.15), raising the possibility of an association at higher titers, limited by the small subgroup and wide confidence interval.

## Discussion

In this prospective study of 176 children with newly diagnosed severe ITP, ANA testing at diagnosis provided no prognostic value for predicting chronic disease, IVIg treatment response, or spontaneous recovery. ANA was present in 20% of children at diagnosis, a 4-fold higher than healthy controls, but was equally common among those who recovered (32/155, 21%) and those with chronic ITP (4/21, 19%). Furthermore, ANA testing added no value beyond the Childhood ITP Recovery Score, a validated clinical prediction tool incorporating age, sex, disease onset, infection history, and platelet count. These findings challenge the practice of routine ANA testing in newly diagnosed ITP and suggest that previous reports of an association may have been confounded by patient selection, treatment effects, or insufficient power.

While we cannot formally exclude a small effect, the consistency of null findings across all analyses argues against a clinically meaningful association being masked by sample size. We observed nearly identical outcomes between ANA-positive and ANA-negative groups, and no trend toward an association with chronic ITP effect in aggregate or subgroup analyses. A high-titer (≥1:160) subgroup was too small to support inferences. Collectively, these findings suggest that any association between ANA and chronic ITP is either absent or too small to guide clinical decision-making for individual patients.

While ANA have been suggested as a marker of chronic ITP in children, conflicting evidence exists regarding the potential benefit as prognostic biomarker. The 20% ANA prevalence at diagnosis, a 4-fold higher than healthy controls, suggests immune dysregulation in newly diagnosed ITP, but this did not translate to prognostic value. We found no association between ANA and antiplatelet antibodies ( Table 2 ), suggesting ANA and platelet-directed autoimmunity are distinct phenomena in childhood ITP. This is consistent with the complex, multifactorial pathophysiology of ITP.

### Previous Studies

The 20% ANA prevalence we observed falls within the 9–44% range reported in previous pediatric ITP studies. 9 11 12 13 Methodological differences may explain discordant findings regarding chronic ITP risk. First, regarding patient selection, previous studies often tested ANA at physician discretion based on concerning features (e.g., adolescent females, insidious onset, family history of autoimmunity), creating selection bias toward higher-risk patients with chronic ITP rates of 52–78%, 13 19 20 21 compared with our unselected cohort (12% chronic rate). Second, regarding timing of testing, several prior studies collected sera weeks to months after diagnosis and after immunomodulatory treatment, 11 13 19 20 some of which can induce false-positive ANA. 18 In contrast, our study design ensured serum collection within 72 h of diagnosis before any treatment. Third, regarding the definition of chronic ITP, most previous studies used the older definition (<150 × 10 ^9^ /L at 6 months) rather than current international consensus (<100 × 10 ^9^ /L at 12 months). We analyzed both definitions and found no association with either ( **Supplementary Table S1** (online only)). Fourth, regarding statistical power, with chronic ITP rates of 10–15%, even our multicenter prospective study had limited power for definitive conclusions. The previous meta-analysis suggesting OR 2.9 8 included only three small retrospective studies with the limitations noted earlier. Our study design, including prospective enrollment, unselected patients, standardized blood sampling times, pre-treatment sampling, and complete follow-up, addresses previous limitations and provides substantial evidence indicating that ANA at diagnosis does not predict chronic ITP.

An important clinical question is whether ANA-positive primary ITP represents early-stage secondary autoimmune disease, particularly SLE. 13 Published data are conflicting. One study found 12/13 ANA-positive children were reclassified as secondary ITP within 2 years, 22 while another found no autoimmune diagnoses in 33 ANA-positive children followed for 2–7 years. 11 Our study cannot resolve this question, but several observations are relevant. We excluded children with clinical signs of secondary ITP at enrollment, potentially removing those at highest risk. Of children with ANA at the diagnosis, 32/36 (89%) recovered from ITP within 12 months. Future studies should include structured long-term follow-up to determine whether positive ANA at ITP diagnosis predicts subsequent autoimmune disease, which would represent a different clinical question than predicting ITP chronicity. Until such data exist, ANA-positive children should be followed clinically, but do not necessarily require intensive autoimmune screening in the absence of other concerning features.

Finally, the pathophysiology of childhood ITP and adult ITP differs significantly, with a higher rate of post-infectious or post-vaccination cases and an overall lower rate of chronic ITP. 23 Thus, the value of ANA for prognosis in adult ITP should be considered separately.

### Clinical Implications

Our findings have several practical implications for pediatric hematology practice. Routine ANA testing in newly diagnosed ITP with severe thrombocytopenia does not seem justified and could lead to unnecessary laboratory utilization and healthcare costs. Laboratories performing platelet antibody testing would not benefit from adding ANA to their routine ITP workup. Clinical risk stratification showed that ANA added no value beyond clinical assessment in any risk stratum of the Childhood ITP Recovery Score, 7 which incorporates readily available clinical variables (age, sex, onset pattern, infection history, platelet count).

Selective ANA testing might be appropriate. Testing should be reserved for patients with clinical features suggesting secondary ITP or evolving autoimmune disease, such as arthritis, rashes, positive family history, cytopenias beyond thrombocytopenia, or atypical disease features. In such cases, ANA might help to identify children requiring additional follow-up for systemic lupus erythematosus or other autoimmune conditions. Moreover, the timing of testing may matter. Our dataset address only baseline ANA testing at diagnosis. Whether ANA testing at later time points (e.g., 3–6 months in persistent ITP) has prognostic value remains unclear. At these later time points, when transient post-infectious ITP cases would have resolved, ANA might be potentially more informative for persistent autoimmune-related disease.

### Generalizability

Several factors affect the generalizability of our findings. First, our cohort included only children with severe thrombocytopenia (platelet count ≤20 × 10 ^9^ /L), representing approximately 80% of children with newly diagnosed ITP. 15 Whether ANA has prognostic value in the around 20% of children with milder thrombocytopenia (20–100 × 10 ^9^ /L) remains unknown and warrants evaluation. Moreover, childhood ITP differs fundamentally from adult ITP, with higher rates of post-infectious cases and spontaneous recovery. 23 Our findings should not be extrapolated to adult ITP, where other disease mechanisms predominate and ANA may have different implications. In addition, our European cohort may not represent populations with different genetic backgrounds or infection patterns. Finally, all participants were enrolled at general pediatric hospitals, ensuring applicability to general pediatric practice.

### Limitations

We had limited power to detect minor effect sizes, and the study was not designed to perform adequately powered subgroup analyses. However, as mentioned earlier, the consistency of null findings across all analyses argues against a clinically meaningful association being obscured by sample size. We evaluated ANA only at diagnosis, before treatment. Whether ANA persistence or seroconversion at 3 to 6 months has prognostic value remains unexplored. Testing at later time points might identify children with persistent autoimmune-mediated disease. Outcomes were only followed for 12 months, and longer follow-up might reveal secondary autoimmune diagnoses. Finally, serum was unavailable for 24/200 enrolled children (12%), though these did not differ in baseline characteristics, making selection bias within our study unlikely.

## Conclusion

In children with newly diagnosed ITP and severe thrombocytopenia, ANA testing at diagnosis does not predict chronic disease, IVIg treatment response, or spontaneous recovery, and provides no incremental prognostic value beyond the Childhood ITP Recovery Score. These findings do not support routine ANA testing at diagnosis. Consequently, ANA testing should not be routinely performed in newly diagnosed ITP, with selective testing guided by clinical suspicion of autoimmune diseases.

Future research should address: (i) later ANA evaluation, e.g., at 3–6 months after diagnosis; (ii) long-term follow-up studies to determine whether ANA predicts secondary autoimmune disease; (iii) ANA assessment in children with mild-to-moderate thrombocytopenia (20–100 × 10 ^9^ /L); and (iv) ANA association with responses to thrombopoietin (TPO) receptor agonists or glucocorticoids.

## Supplementary Material

Ergänzendes Material Supplementary Material
