# Supplementary material for: Prognostic Re-evaluation of Antinuclear Antibodies in Childhood Immune Thrombocytopenia
Source: TH Open. 2026 Sep 17;10:a29386571. doi: 10.1055/a-2938-6571 (PMC13643730; doi:10.1055/a-2938-6571)
Supplement: Supplementary file 1 [file 10-1055-a-2938-6571_29570350.pdf]

Online Supplement

Prognostic Re-evaluation of Anti-Nuclear Antibodies in Childhood Immune Thrombocytopenia.

Supplementary Table S1. Complete recovery and odds ratios (OR) with 95% confidence intervals (CI) for platelet recovery stratified by ANA status.

	IVIg			Observation		
	ANA- (N = 77)	ANA+ (N=18)	OR (95% CI)	ANA- (N = 63)	ANA+ (N=18)	OR (95% CI)
Week 1	53 (68.8)	14 (77.8)	1.58 (0.51 - 6.04)	11 (17.5)	3 (16.7)	0.93 (0.19 - 3.45)
Week 4	51 (66.2)	10 (55.6)	0.64 (0.22 - 1.85)	23 (36.5)	6 (33.3)	0.87 (0.27 - 2.56)
Week 13	64 (83.1)	13 (72.2)	0.53 (0.17 -1.87)	38 (60.3)	12 (66.7)	1.52 (0.49 - 5.26)
Week 26	66 (85.7)	14 (77.8)	0.58 (0.17 - 2.34)	48 (76.2)	14 (77.8)	1.09 (0.33 - 4.30)
Week 52	69 (89.6)	16 (88.9)	0.93 (0.21 - 6.53)	54 (85.7)	16 (88.9)	1.33 (0.30 - 9.33)
Chronic ITP	8 (10.4)	2 (11.1)	1.08 (0.15 - 4.83)	9 (14.3)	2 (11.1)	0.75 (0.11 - 3.29)

Data are n (%) or odds ratios (OR) with 95% confidence intervals (CI). One patient lacked platelet count data one week and two patients at three months follow-up. Chronic ITP was defined as absence of complete platelet recovery at 12 months.

Supplementary Table S2. Reactivity with Hep-2 nuclear antigens (ANA) at various serum dilutions. Additional samples for dilution were available for 14/36 patients with positive ANA at 1:40. Samples were not diluted further if they were only slightly positive (+/-) at 1:40. Left panel, contingency table for dilution from 1:40 -> 1:160. Right panel, contingency table for dilution from 1:160 -> 1:320. Three samples were negative at dilution 1:160 and no longer shown.

	Dilution 1:40		Dilution 1:160		
**Dilution 1:160**	**++**	**Dilution 1:320**	**++**	**+**	**+/-**
**++**	1	**++**			
**+**	5	**+**	1		
**+/-**	5	**+/-**		5	
**-**	3	**-**			5
